# Phosphorylation by ATR triggers FANCD2 chromatin loading and activates the Fanconi anemia pathway

**DOI:** 10.1016/j.celrep.2023.112721

**Published:** 2023-06-30

**Authors:** Marian Kupculak, Fengxiang Bai, Qiang Luo, Yasunaga Yoshikawa, David Lopez-Martinez, Hannan Xu, Stephan Uphoff, Martin A. Cohn

**Affiliations:** 1 Department of Biochemistry, University of Oxford, Oxford OX1 3QU, UK

**Keywords:** ATR, ICL repair, Fanconi anemia, phosphorylation, FANCD2/FANCI, genome instability, cancer

## Abstract

The Fanconi anemia (FA) pathway repairs DNA interstrand crosslinks (ICLs) in humans. Activation of the pathway relies on loading of the FANCD2/FANCI complex onto chromosomes, where it is fully activated by subsequent monoubiquitination. However, the mechanism for loading the complex onto chromosomes remains unclear. Here, we identify 10 SQ/TQ phosphorylation sites on FANCD2, which are phosphorylated by ATR in response to ICLs. Using a range of biochemical assays complemented with live-cell imaging including super-resolution single-molecule tracking, we show that these phosphorylation events are critical for loading of the complex onto chromosomes and for its subsequent monoubiquitination. We uncover how the phosphorylation events are tightly regulated in cells and that mimicking their constant phosphorylation leads to an uncontrolled active state of FANCD2, which is loaded onto chromosomes in an unrestrained fashion. Taken together, we describe a mechanism where ATR triggers FANCD2/FANCI loading onto chromosomes.

## Highlights

• Identification of 10 SQ/TQ sites in FANCD2 that are phosphorylated by ATR
• Phosphorylation of FANCD2 by ATR activates the FANCD2/FANCI complex
• ATR is required for loading of the FANCD2/FANCI complex onto chromosomes
• Super-resolution microscopy tracking of individual FANCD2 molecules in live cells

## Graphical abstract

**Figure:**
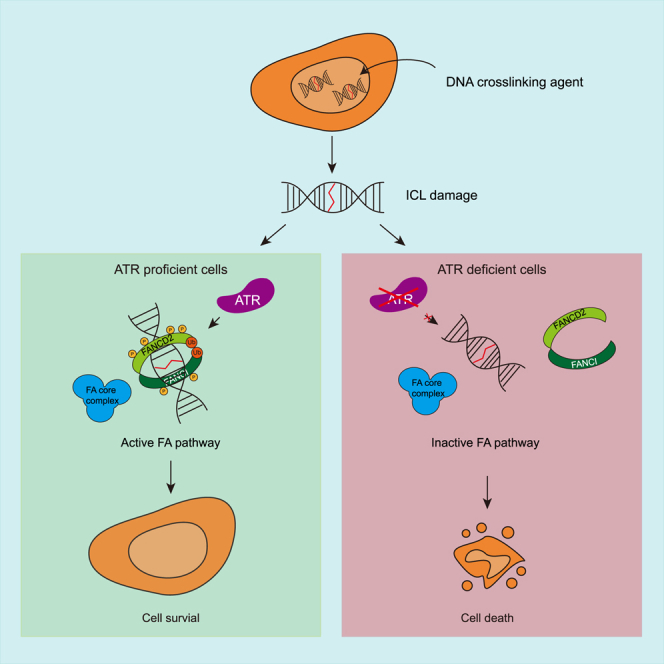

## Teaser

Kupculak et al. identify 10 SQ/TQ sites on FANCD2. ATR phosphorylates these sites to permit chromatin loading of the FANCD2/FANCI complex and activation of the Fanconi anemia pathway. Super-resolution microscopy of individual FANCD2 molecules in live cells demonstrate how genome-wide localization of the FANCD2/FANCI complex depends on ATR.

## Introduction

Cells are facing various DNA damages caused by exogenous and endogenous agents, in which interstrand crosslinks (ICLs) form a covalent bond between two DNA strands caused by diverse environmental and endogenous chemical sources such as endogenously generated aldehydes and the therapeutic agent mitomycin C (MMC).^1^^,^^2^^,^^3^ ICLs are insurmountable obstacles for DNA replication and transcription that require the opening of the two DNA strands and prevent the access of DNA-interacting proteins such as transcription factors and DNA helicases. Thus, ICLs are extremely toxic to the cell and need to be removed promptly and efficiently.^4^ To ensure genomic integrity, cells have developed a complex set of pathways to respond to DNA lesions caused by ICLs, including nucleotide excision repair (NER), translesion synthesis (TLS), and homologous recombination (HR) mediated by the Fanconi anemia (FA)/BRCA repair pathway and the DNA glycosylase NEIL3 pathway.^5^

The FA pathway plays a pivotal role in the repair of ICLs, and defects in the FA pathway result in chromosomal instability leading to congenital abnormalities, bone marrow failure, and cancer susceptibility in humans.^6^ Numerous FA proteins and protein complexes function in the repair process, and to date, 22 FA genes have been identified. The corresponding proteins are involved in a series of critical steps as follows. First, the ICL has to be detected by sensors, such as UHRF1^7^ or stalled replication forks.^5^ Second, the essential FANCD2/FANCI complex is loaded onto chromosomes and subsequently monoubiquitinated by the FA core complex serving as an E3 ligase. Once the FANCD2/FANCI complex is monoubiquitinated, a number of downstream DNA repair factors are recruited sequentially, leading to NER, TLS, and HR, involving key factors such as RAD51(FANCR) and BRCA2(FANCD1).^8^^,^^9^^,^^10^

Monoubiquitination of the FANCD2/FANCI complex while bound on chromosomes stabilizes its interaction with DNA^11^ following a conformational change of the complex.^12^^,^^13^^,^^14^ Because of this key role of the complex, disruption of monoubiquitination of FANCD2 completely abrogates the recruitment of downstream effectors and prevents activation of the FA pathway.^15^ However, the mechanism underlying the licensing of FANCD2 to be loaded onto chromosomes is unclear.

Here, we report the identification of 10 SQ/TQ phosphorylation sites on FANCD2 catalyzed by the ATR kinase. We found that phosphorylation of these sites is greatly increased in cells in response to ICLs and that this event is necessary for activation of the FA pathway. Blocking phosphorylation prevents loading of the FANCD2/FANCI complex onto chromosomes and completely abrogates monoubiquitination of FANCD2. Preventing phosphorylation sensitizes cells to ICL-forming drugs. In good agreement, using live-cell imaging and reconstituted biochemical assays *in vitro*, we demonstrate that constitutive phosphorylation leads to a super-activated form of FANCD2, resulting in a constitutively active FA pathway. The discovered phosphorylation events therefore explain the mechanism underlying licensing of FANCD2 for chromosome loading and activation of the FA pathway.

## Results

### Ubiquitination of FANCD2 is dependent on ATR

**Figure 1:**
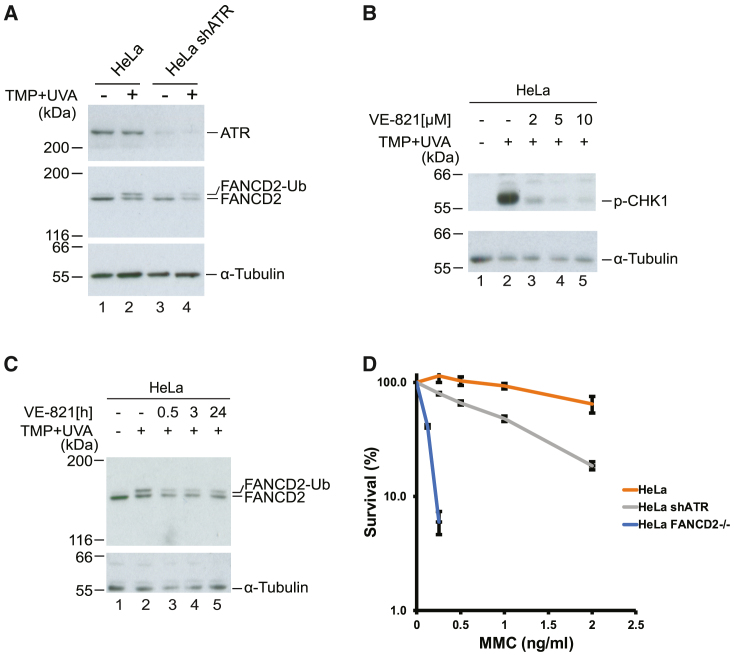
ATR is required for FANCD2 monoubiquitination (A) Immunoblot analysis of HeLa and HeLa + shATR cells upon the introduction of DNA interstrand crosslinks. TMP was added 30 min before irradiation, and the cells were harvested 3 h afterward. (B) Immunoblot analysis of HeLa cells upon the introduction of DNA interstrand crosslinks to test different concentrations of ATR inhibitor (VE-821). VE-821 was added together with TMP 30 min before irradiation, and the cells were harvested 3 h afterward. (C) Immunoblot analysis of HeLa cells upon the introduction of DNA interstrand crosslinks with different VE-821 incubation times before irradiation. VE-821 concentration in all treated samples was 10 μM. TMP was added 30 min before irradiation, and the cells were harvested 3 h afterward. (D) Clonogenic survival assay to determine the viability of HeLa + shATR cells upon the introduction of interstrand crosslinks by MMC (mean ± SEM, n = 3 technical replicates from a representative experiment).

To investigate the role of ATR in the FA pathway, we depleted ATR in HeLa cells. A specific short hairpin RNA (shRNA) was stably expressed, resulting in reduced ATR protein levels compared with control cells (Figure 1A). Upon the introduction of ICLs by treating cells with trimethylpsoralen (TMP) followed by UVA irradiation, a robust monoubiquitination of FANCD2 was observed in control cells, while reduced monoubiquitination was seen in cells where ATR was depleted (Figure 1A, lanes 2 and 4), showing a contribution by ATR to this biological process. To test the observed phenotype under conditions of acute ATR suppression, avoiding depletion of ATR over several days, as is the case with shRNA, we subsequently made use of the specific ATR inhibitor VE-821, which allows the suppression of enzyme activity without affecting the cellular concentration of ATR protein. We first determined the concentration of inhibitor needed to inhibit ATR in the cells used, assessed by phosphorylation of the well-characterized ATR substrate CHK1, in response to ICLs. In the absence of the inhibitor a strong phosphorylation of CHK1 was observed (Figure 1B, lane 2), while the phosphorylation was much reduced in the presence of 10 μM inhibitor (Figure 1B, lane 5). We therefore used this concentration in subsequent experiments. HeLa cells were treated with TMP/UVA to induce ICLs in the presence or absence of the ATR inhibitor, and FANCD2 monoubiquitination was analyzed by immunoblotting. While control cells displayed strong ubiquitination, FANCD2 ubiquitination in cells treated with the ATR inhibitor was attenuated (Figure 1C), again underscoring a role of ATR for activation of FANCD2.

These data show that ATR is necessary for FANCD2 monoubiquitination, suggesting that ATR might be functionally important for the cellular response to ICLs. To address this, we assessed the survival of cells in response to the ICL-inducing drug MMC following depletion of ATR, measured by a clonogenic survival assay. As a control, we used HeLa cells where FANCD2 is entirely absent following disruption of the *FANCD2* gene by CRISPR-Cas9. Consistent with the immunoblotting results, cells with depleted ATR are more sensitive compared with control HeLa cells (Figure 1D).

### Recruitment of FANCD2 to ICLs on chromosomes requires the activity of ATR

**Figure 2:**
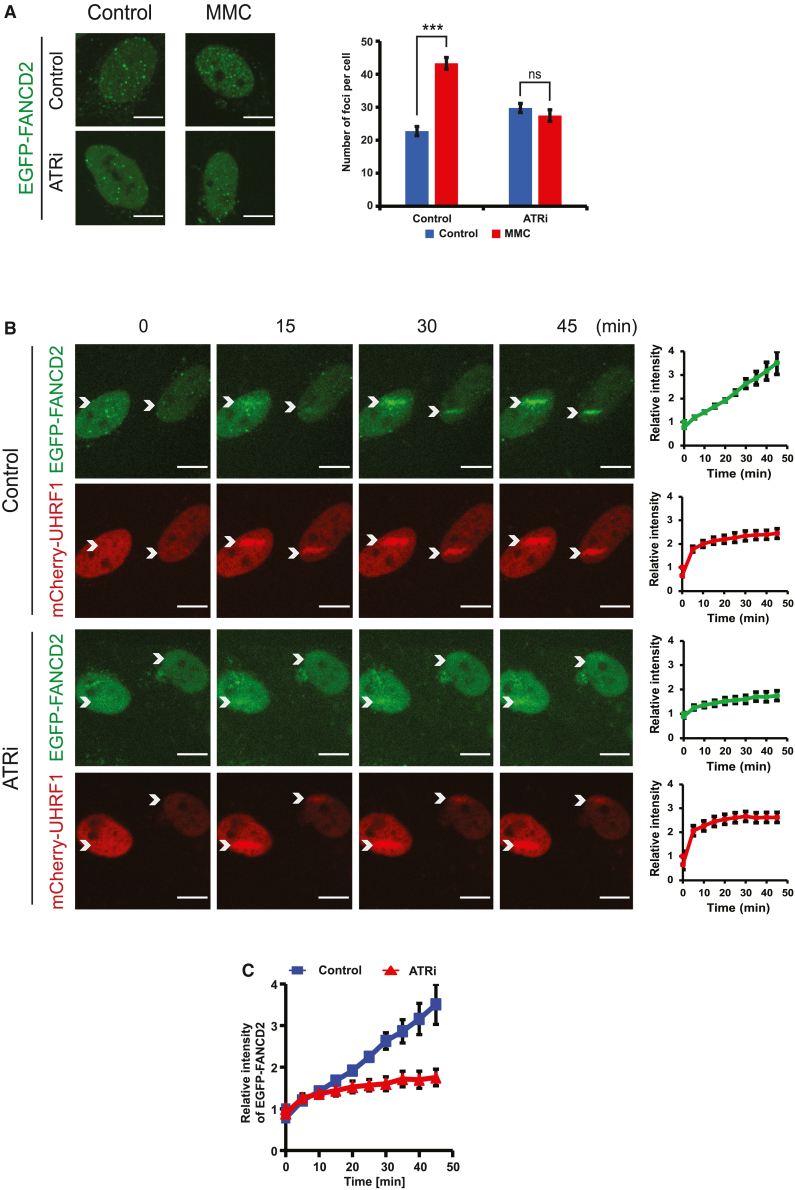
ATR is required for FANCD2 loading onto chromosomes (A) Quantification of FANCD2 foci upon the introduction of ICLs, ± ATR inhibition. VE-821 concentration in all treated samples was 10 μM. The images were taken 16 h after VE-821/MMC treatment. Number of foci quantified as mean ± SEM; number of cells analyzed: 86 for control/no MMC, 78 for VE-821/no MMC, 74 for control/MMC, 82 for VE-821/MMC; n = 2 biological replicates; scale bar, 10 μm. ^∗∗∗^p ≤ 0.001. (B) Live-cell imaging of HeLa *FANCD2*^−/−^ cells complemented with EGFP-FANCD2. After adding TMP, cells were microirradiated at specific areas (marked by white arrows) and recorded for the indicated times. mCherry-UHRF1 was used as the control (stripe intensities quantified as mean ± SEM; number of cells analyzed: 6 for control, 5 for VE-821; n = 3 biological replicates; scale bar, 10 μm). (C) Combination of quantification data from (B), showing recruitment of EGFP-FANCD2 in control cells and in cells where ATR is inhibited. Relative intensities quantified as mean ± SEM. See also Figure S1.

Monoubiquitination of FANCD2 is a critical event for the activation of the FA pathway and takes place only after the FANCD2/FANCI complex has been loaded onto DNA.^11^ Monoubiquitination increases the stability of the complex on DNA, enhancing its retention. Given the finding that ATR is necessary for monoubiquitination, we hypothesized that loading of the complex onto chromosomes would also require ATR. To test this directly in living cells, we stably expressed EGFP-FANCD2 in *FANCD2*^−/−^ cells. We then treated the cells with the ICL-forming agent MMC and assessed FANCD2 foci in live cells, rather than fixed cells, in order to correctly assess the numbers of foci and to avoid potential artifacts resulting from immunofluorescence staining. As expected, the number of EGFP-FANCD2 foci significantly increased in control cells after MMC treatment (Figure 2A). In contrast, the number of EGFP-FANCD2 foci did not increase in response to MMC in cells treated with the ATR inhibitor (Figure 2A), suggesting that ATR is needed for normal recruitment of FANCD2 to ICLs, visualized as nuclear foci.

To test the observations on foci formation using a different method and to gain further insight into the kinetics of recruitment, we then turned to live-cell imaging of cells where ICLs had been introduced by microirradiation in a specific area of the nucleus. To do such experiments, we stably expressed EGFP-FANCD2 in HeLa *FANCD2*^−/−^ cells, together with mCherry-UHRF1, serving as a marker of ICLs (Figure S1A). Following the specific introduction of ICLs, both EGFP-FANCD2 and mCherry-UHRF1 quickly and robustly enriched at ICLs on chromosomes. In stark contrast, when ATR was inhibited, the recruitment of EGFP-FANCD2 was nearly completely abrogated (Figures 2B and 2C). Interestingly, the recruitment of the ICL sensor protein mCherry-UHRF1 was not affected by the inhibition of ATR, suggesting that this process does not depend on such enzymatic activity.

Taken together, these experiments demonstrate that cellular ATR kinase activity is critical for FANCD2 recruitment to chromosomes and for the monoubiquitination of FANCD2. Both events are essential initial steps required to activate the FA pathway.

### Identification of SQ/TQ phosphorylation sites on FANCD2

**Figure 3:**
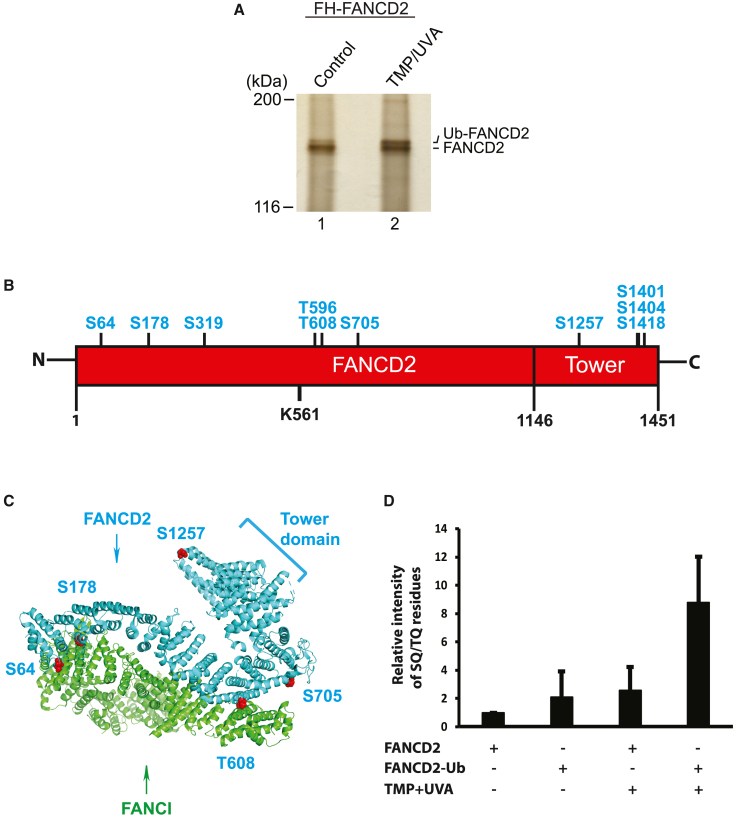
Identification of ATR phosphorylation sites on FANCD2 (A) Silver staining of FLAG-HA-FANCD2 purified from untreated cells (lane 1) and from cells treated with TMP/UVA (lane 2) cells. (B) Location of the 10 SQ/TQ residues that have been found phosphorylated in our mass spectrometry analysis. S222, T691, and S717 previously described in the literature are not shown for clarity. (C) The spatial localization of the mapped S64, S178, T608, S705, and S1257 residues in the structure of FANCD/FANCI complex (PDB: 6AVD) is shown as red spheres. Residues S319, T596, S1401, S1404, and S1418 are not visible in the structure. (D) Relative intensities of the phosphorylated SQ/TQ residues. Each column represents the four different species of bands from silver-stained SDS-PAGE gels (as shown in A) of purified FANCD2 from HeLa cells (FANCD2, no TMP; monoubiquitinated FANCD2 (FANCD2-Ub)/no TMP; FANCD2, TMP; monoubiquitinated FANCD2 (FANCD2-Ub), TMP). Relative intensities of phosphorylated peptides to unmodified peptides as identified by tandem mass spectrometry (MS/MS) in the four samples. Mean of SEM ± SD in n = 2 independent experiments.

The functional importance of ATR for FANCD2 monoubiquitination and recruitment prompted us to further uncover the underlying mechanism. The observed relationship between ATR and FANCD2 could be direct via phosphorylation of FANCD2 itself or indirect. To test this in a comprehensive and unbiased way, we decided to first identify all actual ATR phosphorylation events on FANCD2. Using CRISPR-Cas9 genome engineering, we fused the FLAG-hemagglutinin (HA) epitopes in frame with FANCD2 in HeLa cells.^16^ The obtained cell line allowed us to purify homogeneous FLAG-HA-FANCD2 from HeLa cells via a series of biochemical purification steps. By purifying FANCD2 both from unperturbed cells and from cells in which we had introduced ICLs via TMP/UVA treatment, we could obtain both the unubiquitinated and the monoubiquitinated forms of FANCD2 as observed by silver stain of the proteins separated by SDS-PAGE (Figure 3A). We excised the two species of FANCD2 and subjected them to tandem mass spectrometry (MS/MS) analysis. We identified a number of phosphorylation sites that match the consensus ATR substrate sequence of SQ/TQ, as well as some that were previously reported^17^ (Figure 3B). Careful examination of the positions of the phosphorylated sites, mapped onto the high-resolution cryoelectron microscopy (cryo-EM) structure of the FANCD2/FANCI complex,^12^ showed that many of the phosphorylation sites are located on or near the surface of the complex (S64, S178, T608, S705, and S1257) in its non-ubiquitinated state (Figure 3C). Interestingly, several residues (S64, T608, and S705) are very near flexible areas on the surface of the protein, areas that were not resolved in the structure, suggesting flexibility, typical for phosphorylation sites. The remaining sites are located inside these flexible areas (S319, T596, S1401, S1404, and S1418).

Since we analyzed the phosphorylation status of FANCD2 both before and after ubiquitination, we could assess whether phosphorylation of the SQ/TQ sites increased or decreased dependent on the ubiquitination status of FANCD2. This analysis revealed that the degree of phosphorylation of the SQ/TQ sites on FANCD2 was greater on monoubiquitinated FANCD2 in cells where ICLs had been introduced into the chromosomes (Figure 3D), suggesting an activatory role.

### ATR phosphorylation of FANCD2 promotes its monoubiquitination

**Figure 4:**
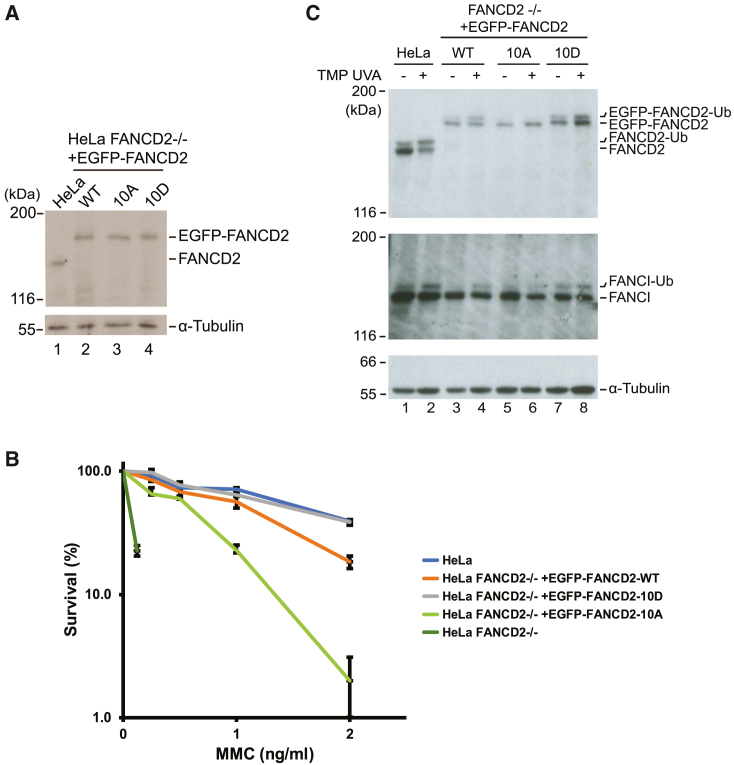
ATR phosphorylation of FANCD2 is required for resistance to MMC in human cells (A) Immunoblot analysis of HeLa cells and the selected clones of HeLa *FANCD2*^−/−^ cells complemented with EGFP-FANCD2-wild type (WT), EGFP-FANCD2-10A (10A), and EGFP-FANCD2-10D (10D). (B) Clonogenic survival assay to determine the viabilities of HeLa *FANCD2*^−/−^ cells complemented with EGFP-FANCD2, EGFP-FANCD2-10A and EGFP-FANCD2-10D (mean ± SEM, n = 3 technical replicates from a representative experiment). (C) Immunoblot analysis of HeLa and HeLa *FANCD2*^−/−^ cells complemented with EGFP-FANCD2, EGFP-FANCD2-10A, and EGFP-FANCD2-10D. After the introduction of ICLs, cells were harvested at the indicated time points. See also Figure S1.

To gain mechanistic insight into the activatory role of FANCD2 phosphorylation, we decided to create two mutant derivatives of the EGFP-FANCD2 protein. In one derivative, we mutated the 10 phosphorylation sites (S64, S178, S319, T596, T608, S705, S1257, S1401, S1404, and S1418) into alanines, and this mutant we call EGFP-FANCD2-10A. The other derivative consisted of a mutant where we changed the 10 residues to aspartic acid to mimic the phosphorylated state of serines and threonines. This mutant we call EGFP-FANCD2-10D. The two mutants, as well as wild-type EGFP-FANCD2, were then stably expressed in HeLa *FANCD2*^−/−^ cells at levels identical to endogenous FANCD2 (Figure 4A). To assess whether either blocking phosphorylation (EGFP-FANCD2-10A) or mimicking phosphorylation (EGFP-FANCD2-10D) impacts FANCD2 activity and the FA pathway, we subjected the cell lines to a clonogenic survival assay using increasing concentrations of MMC. As expected, HeLa *FANCD2*^−/−^ cells were extremely sensitive to MMC compared with HeLa cells (Figure 4B). Expression of EGFP-FANCD2 nearly fully complemented the cells back to the level of resistance observed in HeLa cells. By contrast, cells complemented with EGFP-FANCD2-10A were significantly more sensitive, showing that lack of phosphorylation of FANCD2 weakens its activity. On the other hand, expression of EGFP-FANCD2-10D fully complemented the cell viability, reinforcing that phosphorylation activates the protein complex. Importantly, this mutant, which is mimicking the constitutively phosphorylated state of FANCD2, appears to provide a stronger resistance to MMC than wild-type FANCD2 itself, suggesting that this form of the protein might possess higher activity (Figure 4B).

To gain further insight into the underlying mechanism, we decided to assess the potential of the FANCD2 derivatives to be monoubiquitinated. We treated cells with TMP/UVA to induce ICLs and analyzed cell lysates by immunoblotting. As expected, endogenous FANCD2 and EGFP-FANCD2 were ubiquitinated normally in response to ICLs (Figure 4B, lanes 1–4). EGFP-FANCD2-10A, on the other hand, was not ubiquitinated at all (Figure 4B, lanes 5–6). EGFP-FANCD2-10D, mimicking the phosphorylated state, was ubiquitinated well and in fact appeared to be ubiquitinated at a higher level compared with EGFP-FANCD2 (Figure 4B, lanes 7–8). The super-active state of EGFP-FANCD2-10D is in good agreement with the clonogenic survival data (Figure 4B). Assessing the ubiquitination status of endogenous FANCI revealed an identical pattern to what we observed for FANCD2, suggesting that in cells, FANCI activation follows, and depends on, FANCD2 phosphorylation (Figure 4C). In other words, these findings suggest a hierarchal order of activation of the FANCD2/FANCI complex wherein FANCD2 is first phosphorylated, followed by activation of FANCI.

### Phosphorylation of FANCD2 by ATR promotes its recruitment to chromosomes

**Figure 5:**
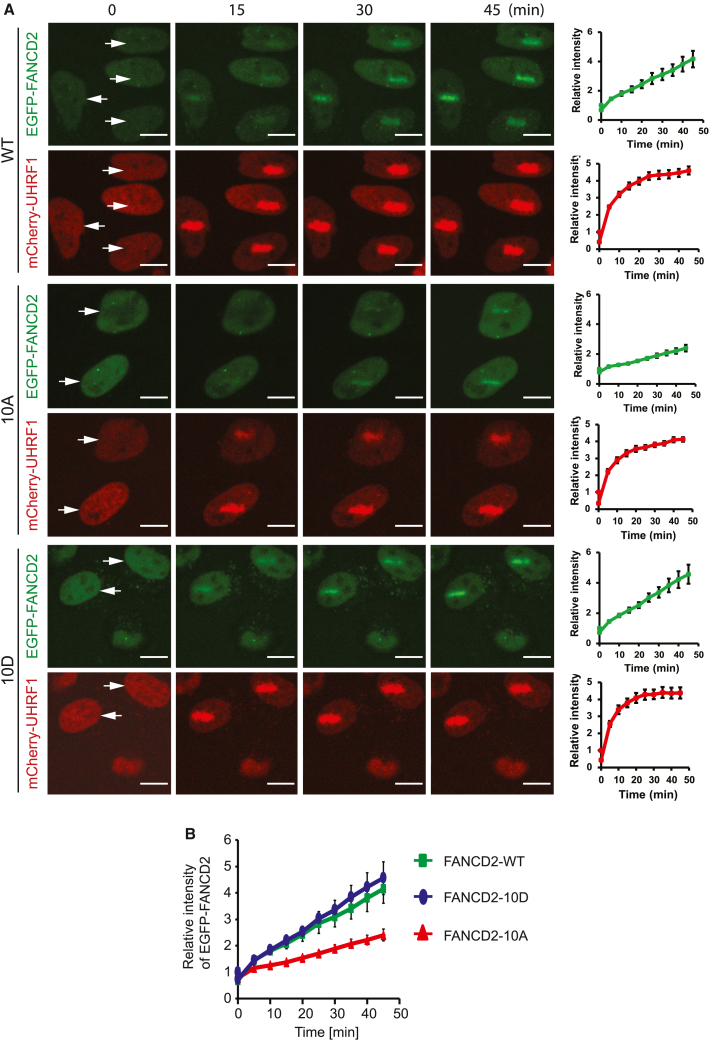
Specific phosphorylation of FANCD2 is required for its recruitment to chromosomes in live cells (A) Live-cell imaging of HeLa *FANCD2*^−/−^ cells complemented with EGFP-FANCD2-WT, EGFP-FANCD2-10A, and EGFP-FANCD2-10D. After adding TMP, cells were microirradiated at specific areas (marked by white arrows) and recorded for the indicated times. mCherry-UHRF1 was used as control (stripe intensities quantified as mean ± SEM; number of cells analyzed: 16 for WT, 11 for 10A, 16 for 10D; n = 3 biological replicates; scale bar: 10 μm). (B) Chart comparing the recruitments of EGFP-FANCD2 for the three analyzed cell lines. See also Figure S1.

Our data show that phosphorylation of FANCD2 by ATR facilitates monoubiquitination and that this, in turn, is important for a functional FA pathway. Given the clear effect on monoubiquitination, we speculated that the actual recruitment to ICLs on chromosomes would also be dependent on the identified ATR phosphorylation sites. To determine this directly, we introduced ICLs in local nuclear regions in live cells expressing the two FANCD2 mutant derivatives alongside mCherry-UHRF1 (Figure S1) and monitored recruitment of these fluorophore-tagged proteins using live-cell imaging (Figures 5A and 5B). Consistent with our hypothesis, and with the monoubiquitination results, both EGFP-FANCD2 and EGFP-FANCD2-10D were recruited efficiently to ICLs (Figures 5A and 5B). In contrast, the recruitment of EGFP-FANCD2-10A was severely reduced. mCherry-UHRF1 was recruited equally well in all three cell lines, showing that the amounts of ICLs introduced were comparable in the three cell lines (Figures 5A and 5B).

Together, these observations support the notion that phosphorylation of FANCD2 on the identified residues promotes its recruitment to ICLs in chromosomes and its subsequent monoubiquitination and, in turn, licenses the activation of the FA pathway.

### ATR is augmenting ubiquitination of FANCD2 and not reducing its deubiquitination by USP1/UAF1

**Figure 6:**
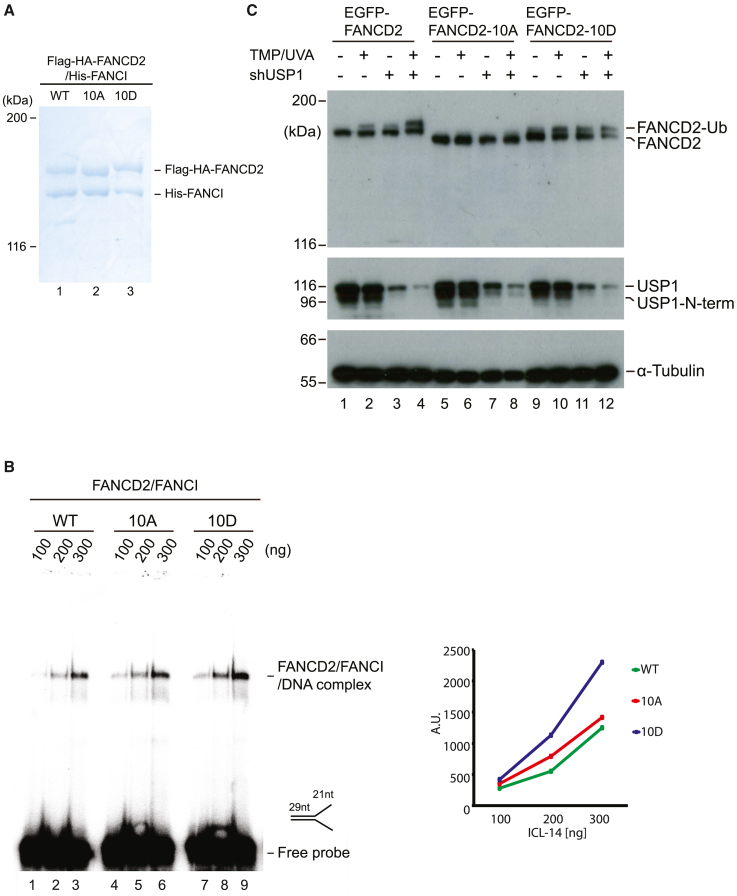
Constitutive phosphorylation of FANCD2 enhances its DNA-binding activity (A) FANCD2/FANCI complexes purified from Sf9 insect cells used for the biochemical assays. Coomassie brilliant blue stained gel. (B) Electrophoretic mobility shift assay (EMSA) showing the DNA-binding potential of the His-FANCI/FLAG-HA-FANCD2 (WT, 10A, or 10D) to a Y-shaped radiolabeled DNA probe (ICL14). Quantification showing intensities of the protein/DNA complexes. (C) Immunoblot analysis of HeLa and HeLa *FANCD2*^−/−^ cells complemented with EGFP-FANCD2, EGFP-FANCD2-10A, and EGFP-FANCD2-10D with or without knockdown USP1 by shRNA. After the introduction of ICLs, cells were harvested at the indicated time points. See also Figures S2 and S3.

Monoubiquitination of FANCD2 requires the preceding loading of the FANCD2/FANCI complex onto DNA. We previously identified a cluster of CK2 phosphorylation sites on FANCD2 that, when phosphorylated, prevent DNA loading via electrostatic repulsion.^16^ Therefore, we speculated that ATR phosphorylation might also affect the DNA binding of the FANCD2/FANCI complex. We created the same two sets of mutations in FANCD2 as already described (10A and 10D) but now as fusion proteins with the FLAG-HA epitope. We then purified these proteins to homogeneity from Sf9 cells as complexes with His-FANCI, leading to the FANCD2/FANCI, FANCD2-10A/FANCI, and FANCD2-10D/FANCI complexes (Figure 6A). Using these three protein complexes, we next assessed their DNA-binding activity to a radiolabeled DNA structure mimicking a stalled replication fork using an *in vitro* electrophoretic mobility shift assay (EMSA). All three complexes were able to interact with DNA (Figure 6B); however, the FANCD2-10D/FANCI complex interacted more strongly than the other two complexes (Figure 6B). All three complexes could be monoubiquitinated in a reconstituted *in vitro* ubiquitination assay (Figure S2).

The observed absence of monoubiquitination of FANCD2 in live cells when ATR phosphorylation is prevented (Figure 4C) could be a result of decreased monoubiquitination or of increased deubiquitination. Deubiquitination of FANCD2 is mediated by the USP1/UAF1 complex.^18^ To gain further mechanistic insight, we decided to test whether ATR phosphorylation controls deubiquitination. To achieve this, we depleted USP1 in HeLa *FANCD2*^−/−^ cells stably expressing EGFP-FANCD2, EGFP-FANCD2-10A, and EGFP-FANCD2-10D and assessed the impact on the monoubiquitination status of these proteins.

As expected, depleting USP1 led to an increase of monoubiquitinated EGFP-FANCD2 (Figure 6C, lane 3), which was further increased when ICLs were introduced in cells (Figure 6C, lane 4). In contrast, the EGFP-FANCD2-10A mutant, which cannot be phosphorylated by ATR, was not monoubiquitinated under any condition (Figure 6C, lanes 5–8). These data show that ATR is promoting monoubiquitination of FANCD2 in live cells to initiate ICL repair rather than playing a role in its deubiquitination. Importantly, the EGFP-FANCD2-10D mutant, which mimics a constitutively phosphorylated state, appears in a super-activated state, with increased monoubiquitination under all conditions (Figure 6C, lanes 9–12).

### Constitutive phosphorylation of FANCD2 deregulates its activity

The observed excessive monoubiquitination of the EGFP-FANCD2-10D mutant suggests that a tightly regulated ATR phosphorylation of FANCD2 is necessary for a functional FA pathway. If that is the case, one would expect that uncontrolled phosphorylation and activation of EGFP-FANCD2-10D would be accompanied by unrestrained loading onto chromosomes in live cells. To test this directly, we assessed the recruitment of the two mutants of FANCD2 to ICLs over a longer time course using live-cell imaging. In agreement with previous observations (Figure 5A), at early time points, EGFP-FANCD2-10A was recruited more weakly than EGFP-FANCD2, while EGFP-FANCD2-10D was recruited slightly more strongly (Figures S3A and S3B). However, at later time points, while both EGFP-FANCD2 and EGFP-FANCD2-10A plateaued, EGFP-FANCD2-10D continued to load onto chromosomes with an even higher rate than at early time points (Figures S3A and S3B). Taken together, these data show that ATR phosphorylation of FANCD2 is necessary and critical for the monoubiquitination of FANCD2 and for the activation of the FA pathway and that this phosphorylation must be controlled to avoid uncontrolled chromatin loading of the FANCD2/FANCI complex.

### Super-resolution single-molecule tracking shows how ATR controls genome-wide localization of FANCD2

**Figure 7:**
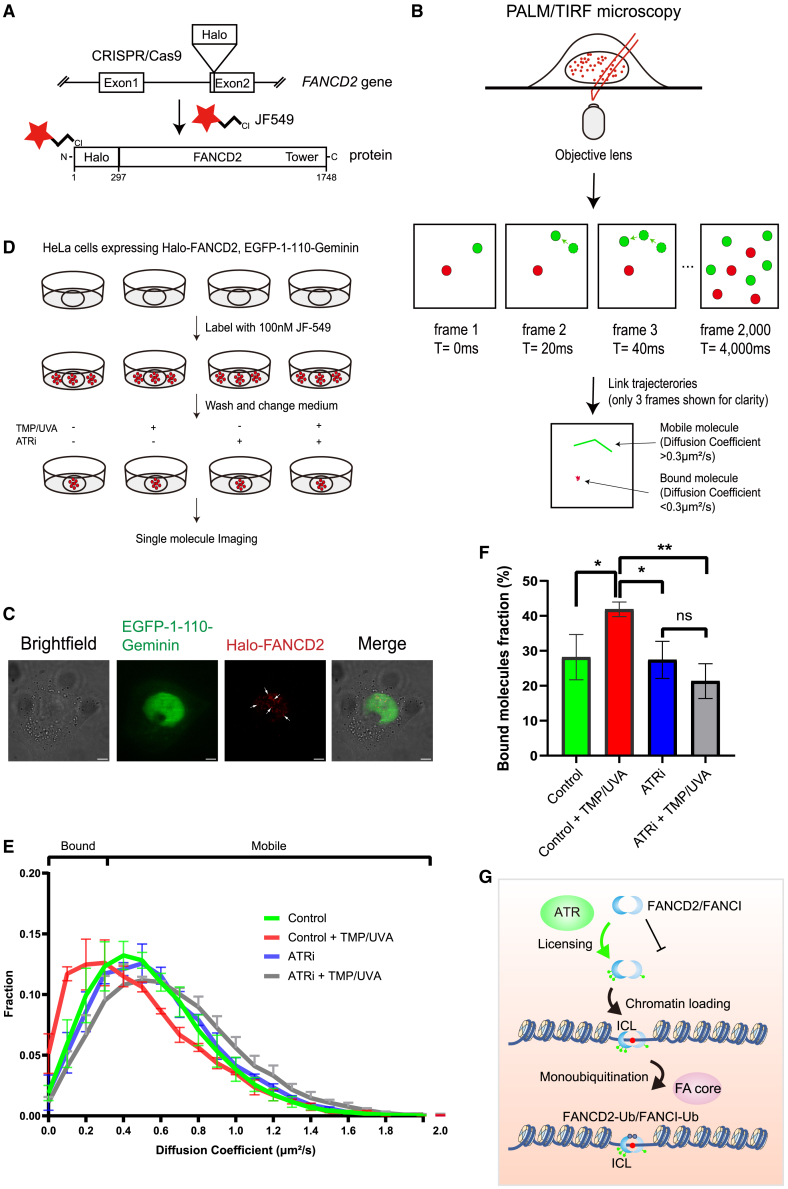
Super-resolution single-molecule tracking shows how ATR controls genome-wide localization of FANCD2 (A) Schematic representation showing the generation of a Halo-FANCD2 knockin HeLa cell line using CRISPR-Cas9. The HaloTag was integrated into the reading frame at the ATG start codon located in exon 2 of the *FANCD2* gene via gene editing. Full HaloTag-FANCD2 knockin cells were then stably transfected with EGFP-1-110-geminin. (B) Schematic of single-molecule tracking experiment. Typically 2,000–4,000 frames of movies with a 20 ms exposure time were acquired to study the movement and chromatin binding of FANCD2 molecules. (C) Super-resolution microscopy images show nuclear EGFP-1-110-geminin and Halo-FANCD2 molecules. S/G2-phase cells with strong EGFP signal were selected and imaged (scale bar, 5 μm). (D) Design of experiments shown in (E) and (F). The non-treated dish was used to determine the natural mobility of FANCD2 molecules. The other three dishes were used to investigate FANCD2 mobility under ICL damage in WT cells, FANCD2 mobility in ATRi-treated cells, and FANCD2 mobility following ICL damage in ATRi-treated cells, respectively. The ATR inhibitor VE-821 (ATRi) was used. Cells were treated with 2 μM ATRi and 4 μg/mL TMP for 1 h before imaging. (E) Distribution of diffusion coefficients of FANCD2 molecules that possess at least 5 localizations per track. The data are split into two populations based on the diffusion coefficient per track, bound FANCD2 molecules (D ≤ 0.3 μm^2^/s), and mobile FANCD2 molecules (D > 0.3 μm^2^/s) (4 cells per treatment, > 5,000 tracks per cell, n = 2 biological replicates). Data are means ± SD. (F) Fraction of bound FANCD2 molecules in each treatment. Data are means ± SD. ^∗^p ≤ 0.05 and ^∗∗^p ≤ 0.01 (ANOVA). G) Model of ATR-dependent FANCD2 activation. Phosphorylation of FANCD2 by ATR permits loading of the FANCD2/FANCI complex onto chromosomes. Once the complex is loaded, monoubiquitination by the FA core complex can take place. See also Figure S4.

Our data so far show how ATR licenses FANCD2 for chromatin loading via phosphorylation of specific sites on FANCD2. We have shown this using various complementary experimental approaches, including reconstituted biochemical reactions *in vitro*, and genetics and other cell biological approaches, for instance live-cell imaging *in vivo*. Common for all these powerful approaches is that they are based on measurements of an average of many molecules, whether *in vitro* or *in vivo*. To gain further insight into the mechanism of how ATR activates FANCD2, we therefore sought to establish and use a single-molecule imaging system *in vivo*. Using CRISPR-Cas9 knockin, we created a cell line expressing FANCD2 fused to the HaloTag (Figure 7A). The HaloTag can then be labeled using the bright synthetic fluorophore JF549, permitting the visualization of individual FANCD2 molecules in live cells using super-resolution localization microscopy. In this setting, JF549 fluorophores randomly switch between a fluorescent and a long-lived non-fluorescent state such that we can record the positions and movement of hundreds to thousands of individual fluorescent FANCD2 proteins per cell over time by taking images at intervals of 20 ms per frame (Figure 7B). After linking molecule localizations to tracks using automated software, we can compute the apparent diffusion coefficients (D, μm^2^/s) from the average displacement of each observed molecule. This approach allows quantifying the extent of DNA binding of a protein in live cells by distinguishing tracks with a low diffusion coefficient (corresponding to an immobile, or bound, molecule) from those with a high diffusion coefficient (corresponding to a mobile, or free, molecule). To determine the threshold between immobile and mobile molecules, we first measured the distribution of diffusion coefficients in cells that had been extensively fixed with paraformaldehyde, ensuring that essentially all FANCD2 molecules are immobile (Video S1; Figure S4A). We observed a distribution of diffusion coefficients ranging from 0 to about 1 μm^2^/s, though the vast majority of molecules had coefficients below 0.3 μm^2^/s (Figures S4B and S4C). We then established a cell line stably expressing free Halo-NLS protein at comparable levels to that of Halo-FANCD2 (Figure S4D). The NLS peptide was included to ensure a strict nuclear localization (Figure S4E). Using this cell line, we measured the distribution of diffusion coefficients of free Halo-NLS to range from 0 to about 2.5 μm^2^/s, with most molecules having coefficients well above 0.3 μm^2^/s (Video S2; Figure S4F). Therefore, in the following experiments, we classified molecules with a diffusion coefficient of 0.3 μm^2^/s or less as immobile/bound and molecules with coefficients above 0.3 μm^2^/s as mobile (Figure S4G).

Video S1. Timelapse of fixed cell expressing Halo-FANCD2 shown in Figure S4A, related to Figure S4 Time between each frame is 20 ms. The total time of recording is 10 s. Scale bar, 5 μm.

Video S2. Timelapse of cell expressing Halo-NLS shown in Figure S4B, related to Figure S4 Time between each frame is 20 ms. The total time of recording is 10 s. Scale bar, 5 μm.

FANCD2 and the FA pathway are primarily active in the S/G2 phase of the cell cycle.^2^ Thus, to obtain even greater precision of our biological measurements, we stably expressed a cell-cycle marker, the 110 N-terminal amino acids of geminin fused to EGFP, a fusion protein that is present in S/G2 cells but is absent in G1 cells.^19^ With this setup, we could easily identify S/G2-phase cells as well as individual nuclear FANCD2 molecules (Figure 7C). In these live cells, we observed individual FANCD2 molecules in cell nuclei, ranging from completely immobile to highly mobile (Video S3). Quantification of these molecules revealed a range of diffusion coefficients from 0 to 2 μm^2^/s (Figure 7E, green curve). 28% of the FANCD2 molecules are bound to chromosomes in this unperturbed state.

Video S3. Timelapse of a control cell expressing Halo-FANCD2 used in Figures 7E and 7F, related to Figure 7 Time between each frame is 20 ms. The total time of recording is 10 s. Scale bar, 5 μm.

Using this system, we then specifically introduced ICLs into the genome by TMP/UVA and quantified the population of bound FANCD2 molecules compared with untreated control cells. The fraction of immobile FANCD2 molecules increased significantly after the introduction of ICLs (Figures 7E and 7F, compare green and red curves). Compared with 28% bound FANCD2 molecules, we now observed approximately 42% of the molecules bound to chromosomes 15 min after the introduction of ICLs. These data represent the beginning of single-molecule observations of FA proteins in living cells. We then went on to treat cells with the ATR inhibitor VE-821 to determine whether the genome-wide loading of FANCD2 to ICLs in chromosomes is affected. Upon inhibiting ATR, we observed a complete abrogation of loading of FANCD2 in response to TMP/UVA, evidenced by a lack of tracks with very low diffusion coefficients (Figures 7E and 7F, compare blue and gray curves). Taken together, these super-resolution single-molecule tracking data in live cells show that phosphorylation of FANCD2 by ATR is crucial for its licensing for chromatin loading and for the activation of the FA pathway.

## Discussion

In this study, we identified 10 SQ/TQ phosphorylation sites on FANCD2 that, when phosphorylated by ATR, license FANCD2 recruitment to ICLs on chromosomes and trigger its following activatory monoubiquitination. Correspondingly, the relative amount of these phosphorylation events was increased in monoubiquitinated FANCD2 in cells treated with ICL-inducing drugs compared with non-ubiquitinated FANCD2 in untreated cells. Cells expressing a non-phosphorylated mutant, FANCD2-10A, displayed increased sensitivity against MMC, no monoubiquitination, and reduced recruitment of FANCD2 to chromatin. In contrast, the phosphorylation-mimicking mutant FANCD2-10D demonstrated increased resistance to MMC, enhanced monoubiquitination, and excessive chromatin loading. These findings show that the discovered SQ/TQ phosphorylation sites are required for FANCD2 activation and recruitment to ICL sites. Moreover, we found that DNA-binding activity of the phosphorylation-mimicking mutant FANCD2-10D was increased *in vitro* compared with FANCD2, showing that phosphorylation of FANCD2 on the SQ/TQ sites leads to increased DNA affinity. *In vivo,* this resulted in increased monoubiquitination and retention at the ICL sites on chromosomes.

These findings are in good agreement with previous studies suggesting a contribution of ATR to ICL repair.^17^^,^^20^^,^^21^^,^^22^ The identified SQ/TQ phosphorylation sites on FANCD2 that are phosphorylated after ICLs are introduced are phosphorylated primarily by ATR, although a minor contribution by other DNA damage response kinases is possible, such as ATM or DNA-PKcs. ATM and DNA-PKcs are activated primarily in response to DNA double-strand breaks,^23^ while ATR is the main kinase activated in response to ICLs.^10^ It is likely that there is some overlap in sites phosphorylated by ATM after X-ray irradiation^24^ and the 10 SQ/TQ phosphorylation sites phosphorylated in response to ICLs. However, considering the presented findings, it is likely that ATR is the major kinase for the 10 SQ/TQ sites after induction of ICLs. Once ATR phosphorylates FANCD2, the DNA affinity of the FANCD2/FANCI complex increases, licensing it for loading onto ICLs residing on chromosomes. Once bound to DNA, the complex can now be monoubiquitinated by the FA core complex and fully activated (Figure 7G). By tracking the motion of individual proteins in the nuclei of live cells using super-resolution single-molecule imaging, we were able to visualize the behavior of individual FANCD2 proteins in living cells. Using this approach, we uncovered that the ATR phosphorylation state of FANCD2 determines its genome-wide loading onto chromosomes in response to introduced ICLs.

### Role of phosphorylation and dephosphorylation in the DNA damage response

The functional importance of ATR is increasingly clear in the DNA damage response, and in addition to FANCD2, ATR has been shown to also phosphorylate the FA core complex and FANCI to activate the FA repair pathway.^17^^,^^22^^,^^25^^,^^26^^,^^27^^,^^28^ Consistent with its activation role for the FA core complex, the 10 ATR phosphorylation sites on FANCD2 are necessary for its activation. By contrast, a different phosphorylation cluster in FANCD2, mediated by CK2, inhibits its function.^16^ On the one hand, phosphorylation of FANCD2 on the CK2 sites causes reduced DNA binding activity and attenuated recruitment to ICL sites and monoubiquitination, and phosphorylation of these sites was reduced in response to ICLs. On the other hand, phosphorylation of FANCD2 on the ATR sites reported in the present work was induced by ICL damage, leading to increased DNA binding, monoubiquitination, and recruitment to ICL sites. Putatively, phosphorylation of the CK2 sites and dephosphorylation of the ATR sites are required to permit unloading of the FANCD2/FANCI complex from chromosomes, thereby shutting down the FA pathway, though more work is needed to clarify this speculation. So, ATR, CK2, and corresponding phosphatases are likely coordinating the FANCD2 activity through its phosphorylation status.

### Phosphorylation and binding of the FANCD2/FANCI complex to DNA before its monoubiquitination

FANCI is known to be phosphorylated by ATR on S556, S559, and S565.^27^^,^^28^^,^^29^ Phosphorylation of S556 facilitates FANCI/FANCD2 monoubiquitination, while phosphorylation of S559 and S565 inhibit USP1/UAF1 deubiquitination. Our non-phosphorylation mutant, FANCD2-10A, completely prevented monoubiquitination of FANCI, demonstrating that phosphorylation of FANCD2 is the initial event that is necessary for FANCI activation in humans and, in turn, for activation of the FANCD2/FANCI complex. These findings are in good agreement with recent data showing that FANCD2 is monoubiquitinated before FANCI.^30^ Simultaneously, data on the chicken homologs suggest that phosphorylation of FANCI is sufficient to activate the FANCD2/FANCI complex *in vitro*.^31^ It is possible that differences in findings can be explained by different species used in the two studies (chicken and humans) and also that, in the former study, all experiments were conducted *in vitro*, while the current study is based primarily on experiments using live cells. Under the USP1-depleted condition, FANCD2-10A- or FANCD2-10D-expressing cells did not demonstrate any increase of monoubiquitination compared with paired cells without USP1 depletion. Thus, ATR phosphorylation of these sites in FANCD2 augments ubiquitination rather than inhibiting deubiquitination. In other words, it appears that FANCD2 phosphorylation status is decisive to the FA pathway, determining the subsequent FANCI ubiquitination that follows.

DNA-binding activity is required for effective FANCD2/FANCI ubiquitination.^16^ The phosphorylation-mimicking mutant FANCD2-10D exhibits increased DNA binding compared with FANCD2 or the FANCD2-10A mutant. Taken together, our data show that phosphorylation of the FANCD2/FANCI complex by ATR increases its DNA-binding activity as well as its monoubiquitination in live cells.

### The role of phosphorylation mediated by ATR in the ubiquitination process

Our data show that the non-phosphorylatable FANCD2-10A nearly abolished the ubiquitination process of both FANCD2 and FANCI, highlighting the functional importance of phosphorylating FANCD2 on these sites for the ubiquitination of both FANCD2 and FANCI mediated by the FA core complex. The structures of the human FA core complex bound to FANCD2/FANCI in different catalytic stages have been solved to near-atomic resolution.^13^^,^^32^^,^^33^ The structural data allow a careful examination of the positions of the ATR phosphorylation sites. S64, S178, T608, S705, and S1257 are visible in FANCD2/FANCI bound to the FA core complex and did not form direct interactions with the FA core in the structure. The S319, T596, S1401, S1404, and S1418 sites were not visible in the structure and thus likely reside in flexible regions. T596 is located on a loop under the ubiquitin-conjugating enzyme UBE2T, and S319 also resides in a loop that is close to the FANCE subunit of the core complex, highlighting the plausible role of the phosphorylation of S319 and T596 for the interaction between the FA core and the FANCD2/FANCI complex. S1401, S1404, and S1418 reside at the C-terminal domain, which is far from the FA core complex. Recently, the cryo-EM structure of chicken FANCD2 in complex with a phosphorylation-mimicking mutant of chicken FANCI was revealed.^31^ The non-phosphorylated chicken FANCD2/FANCI complex can interact with DNA but maintains an open clamp form. However, this clamp is closed in a complex between FANCD2 and a phosphorylation-mimicking FANCI mutant through interactions between the C-terminal tower domain of FANCD2 and the C terminus of FANCI. Thus, it is possible that the three phosphorylation sites (S1401, S1404, and S1418) in this region of FANCD2 might support the interaction with the C-terminal region of FANCI. Whether other residues also play important roles in different catalytic processes and the precise function of phosphorylation on those residues is an outstanding question.

### Conclusions

In this study, we found that ATR plays a key role for activation of FANCD2 via controlling its genome-wide loading onto chromosomes and its subsequent ubiquitination. We identified 10 SQ/TQ phosphorylation sites on FANCD2 that explain this mechanism. Phosphorylation of these sites results in increased DNA-binding activity of the FANCD2/FANCI complex and is necessary for the complex to localize to ICLs on chromosomes. This mechanism explains how the FANCD2/FANCI complex is licensed for chromatin loading in live cells. These findings could provide therapeutic opportunities given the role of the FA pathway in repairing ICLs in chromosomes formed by chemotherapeutic drugs currently used to treat multiple types of cancer.

### Limitations of the study

ATR is the main kinase phosphorylating the 10 SQ/TQ sites in FANCD2 reported in this study. However, other kinases might also act on some or all of these sites. The relative functional contribution of individual sites is currently not known, and we do not know whether all sites on a given FANCD2 molecule are phosphorylated simultaneously. Addressing the latter question is currently not possible with existing technology. Finally, this work leads to a key question, namely the order of events for activation of the FANCD2/FANCI complex. As described in the present work, the complex has to be phosphorylated by ATR on some residues in order to be activated. However, it is also known that CK2 phosphorylation of other residues in FANCD2 inhibits loading of the FANCD2/FANCI complex onto chromosomes, preventing its activation.^16^ Thus, it remains to be determined whether these two events are dependent on each other and in which order they take place in cells.

## STAR★Methods

### Key resources table

**Table:** 

REAGENT or RESOURCE	SOURCE	IDENTIFIER
**Antibodies**		

Mouse monoclonal anti-FANCD2 (FI17)	Santa Cruz Biotechnology	Cat# sc-20022; RRID: AB2278211
Rabbit polyclonal anti-FANCI	FARF	N/A
Mouse monoclonal anti-α-Tubulin (DM1A)	Sigma-Aldrich	Cat# 05-829; RRID: AB310035
Mouse monoclonal anti-FLAG (M5)	Sigma-Aldrich	Cat# F4042; RRID: AB439686
Mouse monoclonal anti-UHRF1 (H-8)	Santa Cruz Biotechnology	Cat# sc-373750; RRID: AB10947236
Rabbit polyclonal Anti-ATR	Abcam	Cat# ab2905; RRID: AB303400
Rabbit monoclonal anti-pCHK1 (Ser345), (133D3)	Cell signaling technology	Cat# 2348; RRID: AB331212
Mouse monoclonal anti-HaloTag	Promega	Cat# G9211; RRID: AB2688011
Rabbit polyclonal anti-USP1	Proteintech	Cat# 14346-1-AP; RRID: AB2214314
Rabbit anti-mouse immunoglobulins (horseradish peroxidase conjugated)	Dako-Agilent	Cat# P0260; RRID: AB2636929
Goat anti-rabbit immunoglobulins (horseradish peroxidase conjugated)	Dako-Agilent	Cat# P0448; RRID: AB2617138

**Bacterial and virus strains**		

Top10	Thermo Fisher	Cat# 404010

**Chemicals, peptides, and recombinant proteins**		

FuGENE6	Promega	Cat# E2691
Lipofectamine 2000	Invitrogen	Cat# 11668019
Geneticin (G418 sulfate)	Thermo Fisher	Cat# 10131-035
VE-821	Sigma-Aldrich	Cat# SML1415
JF549	Promega	Cat# GA1110
Protein A Sepharose Cl-4B	Thermo Fisher	Cat# GE17-0963-02
Dynabeads Goat Anti-Mouse IgG	Thermo Fisher	Cat# 11033
Anti-FLAG M2 agarose resin	Sigma-Aldrich	Cat# A2220
Ni^2^–NTA agarose resin	QIAGEN	Cat# 30210
Trioxsalen (TMP)	Sigma-Aldrich	Cat# 6137
Mitomycin C from *Streptomyces caespitosus*	Sigma-Aldrich	Cat# M4287
Benzonase	Sigma-Aldrich	Cat# E1014

**Critical commercial assays**		

DNA sequencing	Source BioScience	N/A

**Experimental models: Cell lines**		

HeLa FANCD2-/-	Liang et al.^11^	N/A
HeLa FANCD2-/- + EGFP-FANCD2 + mCherry-UHRF1	This paper	N/A
HeLa FANCD2-/- + EGFP-FANCD2-10A + mCherry-UHRF1	This paper	N/A
HeLa FANCD2-/- + EGFP-FANCD2-10D + mCherry-UHRF1	This paper	N/A
HeLa + EGFP-FANCI + mCherry-UHRF1	This paper	N/A
HeLa Flag-HA-FANCD2 knock-in (partial)	This paper	N/A
HeLa Halo-FANCD2 knock-in + EGFP-1-110-Geminin	This paper	N/A

**Recombinant DNA**		

pOZ-N	Nakatani and Ogryzko^34^	N/A
pOZ-N-EGFP-FANCD2	This paper	N/A
pOZ-N-EGFP-FANCD2-10A	This paper	N/A
pOZ-N-EGFP-FANCD2-10D	This paper	N/A
pOZ-N-EGFP-FANCI	This paper	N/A
pBlueScript II SK (+)	Addgene	Cat# 10359-016
pFB-FLAG-HA	Liang et al.^11^	N/A
pFastBac1	Thermo-Fisher	Cat# 10360014
pSpCas9(BB)-2A-Puro (PX459)	Addgene	Cat# 48139
pHTN-HaloTag-3xNLS-CMV-neo	Huseyin et al.^35^	N/A

**Software and algorithms**		

Volocity	Quorum Technologies	N/A
Fiji	Fiji.sc	N/A
PyMOL	Pymol.org	N/A
Matlab R2020b	Mathworks	N/A
Stormtrack	Uphoff et al.^36^	N/A
Graphpad prism 9	Graphpad by Dotmatics	N/A

**Other**		

PE Ultraview Spinning Disk Microscope	Micron Facility, University of Oxford	N/A
SPT microscope (PALM microscope)	Micron Facility, University of Oxford	N/A
Spectrolinker XL-1500 (365 nm)	Department of Biochemistry, University of Oxford	N/A

### Resource availability

#### Lead contact

Further information and requests for resources and reagents should be directed to and will be fulfilled by the lead contact, Martin A. Cohn (martin.cohn@bioch.ox.ac.uk).

#### Materials availability

Materials will be provided without restrictions upon request to lead contact.

### Experimental model and study participant details

#### Cell lines

HeLa, HeLa S3 and Phoenix A cell lines (originally from ATCC) were grown in Dulbecco’s modified eagle medium (D5796, Sigma Aldrich) supplemented with 10% Fetal Bovine Serum (F7524, Sigma Aldrich). Cells were incubated at 37°C and 5% CO_2_ and passaged using Trypsin-EDTA (0.25%, T4049, Sigma Aldrich).

### Method details

#### Plasmids and transfection

EGFP-fused proteins were expressed using the pOZ-N plasmid.^34^ The point mutations were gradually introduced by inverse PCR-based site-directed mutagenesis into the pFB-EGFP-FANCD2 construct and the mutated FANCD2 was subsequently subcloned into the pOZ vector. The final constructs were introduced to cells by retroviral transfection using FuGENE6 (Promega) while following the manufacturer’s protocol. shRNA mediated knock-down of ATR and USP1 were carried out by expressing the target sequences 5’-CCTCCGTGATGTTGCTTGA-3’ and 5’-ACAGTTCGCTTCTACACAA-3’, respectively, from the pSuper.retro vector (Clontech). Halo-NLS was expressed by the pHTN-HaloTag-3xNLS-CMV-neo plasmid, which is a modified version of the pHTN-HaloTag-CMV-neo vector modified with 3x SV-40 NLS at the C-terminus.^35^ 5 μg plasmid DNA was transfected into HeLa cells by Lipofectamine 2000, based on the instructions from the manufacturer. After transfection, cells were selected with 400 μg/ml of G418 in the fully supplemented DMEM medium for two weeks. Resistant cells were seeded for single-clone selection. Expression of the Halo-NLS protein in each clone was tested by immunoblot analysis and fluorescence imaging.

#### Clonogenic survival assay

Cells (400/800/2,000 for HeLa FANCD2^-/-^; 200/400/1,000 for all other cell lines) were plated in six-well plates and treated with different dosages of ICL inducing agents the next day. Colony formation was scored 13-14 days after the treatment using 1% (w/v) crystal violet in methanol.

#### Antibodies

Antibodies used were as follows: anti-FANCD2, 1:100 dilution (sc-20022, Santa Cruz Biotechnology); anti-FANCI, 1:500 dilution (FARF); anti-Flag, 1:1,000 dilution (M5, F4042, Sigma-Aldrich); anti-UHRF1, 1:1,000 dilution (sc-373750, Santa Cruz Biotechnology); anti-α-Tubulin, 1:2,000 dilution (05-829, Merck-Millipore), anti-ATR, 1:100 dilution (ab2905, abcam); anti-pChk (S345), 1:1,000 dilution (133D3, Cell Signaling Technology), anti-USP1, 1:1,000 dilution (14346-1-AP, Proteintech).

#### Whole cell lysate preparation

Cells were scrapped off the dishes, collected into 1.5 ml Eppendorf tubes, spun down at 17,000xg for 5-10sec and washed with PBS. After aspirating supernatants, the pellets were resuspended in equal amount of benzonase buffer (2 mM MgCl_2_, 20 mM Tris pH 8.0, 10% glycerol, 1% Triton X-100 and 12.5 units/ml benzonase (E1014, Sigma)) and left on ice for 10-15 min. To lyse the cells, an equal volume of 2% SDS was subsequently added to each sample which was then heated at 70°C for 2min. The protein concentration was determined using Bradford assay (Bio-Rad Life Science.).

#### Immunoblotting

Protein extracts where mixed with 6x SDS loading buffer (0.375 M Tris pH 6.8, 12% SDS, 60% Glycerol, 0.06% BPB) and DTT was added to a final concentration of 50 mM, topped up with milliQ to achieve concentration 1μg/μl and heated at 95°C for 5-10 min. The samples were loaded into an SDS-polyacrylamide gel along with the Mark 12 Protein Standard (Thermo Fisher Scientific) and run in 1x Running buffer for 2.5-3.5h at 70-100V. Running buffer contained 25mM Tris, 192mM glycine and 0.1% SDS. Separating gel contained -5%-15% acrylamide/bis-acrylamide, 400 mM Tris HCl pH 8.8, 0.1% SDS, 0.1% APS (ammonium persulphate) and 0.1% TEMED (Sigma), stacking gel contained 4% acrylamide/bis-acrylamide, 125 mM Tris HCl pH 6.8, 0.1% SDS, 0.1% APS and 0.1% TEMED. After the run, proteins were transferred onto a nitrocellulose membrane (Amersham) by electroblotting, which was then stained with 1x Ponceau S (VWR Chemicals) dye and the regions of interested were cut out. The membranes were blocked in 5% milk dissolved in 1x TBS-T (150 mM NaCl, 20 mM Tris pH 8.0, 0.1% Tween 20) for 1-3h at the room temperature. Primary antibody incubations were carried out overnight at 4°C in 5% milk complemented with 0.1% sodium azide. On the following day, the membranes were washed three times for 5 min in 1x TBS-T and incubated with secondary antibodies for 1h at the room temperature. After 3 subsequent washes for 7 min in TBS-T the membranes were coated with ECL Western blotting Substrate (Perking Elmer) and developed using X-ray films (Amersham).

#### Mass spectrometric analysis

To identify phosphorylation events in FANCD2 before and after introduction of interstrand crosslinks the protocol proceeded as follows.

Flag-HA-FANCD2 purified from HeLa S3 cells before and after damage was reduced by DTT while cysteine residues were derivatized with iodoacetamide. The proteins were separated by SDS-PAGE and silver stained by using SilverQuest kit (Invitrogen). The specific bands were cut out and in-gel digested with trypsin.^37^ The generated peptide mixtures were subjected to LC-MS/MS using a hybrid linear ion trap/FT-ICR mass spectrometer (LTQ FT, Thermo Electron) described as previously.^38^ MS/MS spectra were assigned by searching them with the SEQUEST algorithm^39^ against the human International Protein Index sequence database.

#### Protein purification

Purification of the FANCD2/FANCI complex or its mutant derivatives including FANCD2-10A/FANCI and FANCD2-10D/FANCI and the proteins for ubiquitination assay was same with the previously reported method.^16^ Briefly, for FANCD2/FANCI complexes and UBA1, Sf9 cells were washed with PBS, harvested, resuspended in lysis buffer (20 mM Tris-HCl pH 8.0, 0.1 M KCl, 10% glycerol, 0.2 mM PMSF. Lysates were clarified by 20 min centrifugation (17,000g), and the collected supernatants were incubated with M2 anti-FLAG agarose resin (A2220, Sigma) at 4°C for 2h. The resin was extensively washed in the original lysis buffer and the proteins were eluted in the lysis buffer complemented with 0.5 mg/ml FLAG peptide. For FANCL, Sf9 cells were washed, harvested and resuspended in detergent-containing lysis buffer (20 mM Tris-HCl pH 8.0, 0.1 M KCl, 10% glycerol, 0.2 mM PMSF and 2 mM β-mercaptoethanol, 0.1% Tween-20) without any sonication. The rest of the purification proceeded the same way as for FANCD2/FANCI complexes and UBA1. 6xHis Ubiquitin was expressed in E. coli BL21(DE3) (Novagen). The cells were grown in Lysogeny Broth supplemented with antibiotics at 37°C until OD600 0.6-0.8 was reached. The expression of 6xHis ubiquitin was then induced by 0.5 mM IPTG and the cells were left to grow overnight at 16°C. Harvested cells were lysed using a french press in the buffer containing 0.5 M NaCl, 0.1 M Tris pH 8, 0.02 M Imidazole and 0.25 mM TCEP. The lysate was then clarified by centrifugation (48,000g) and the supernatant was incubated with Ni^2+^-NTA agarose (QIAGEN) for 2 h at 4°C. Finally, ubiquitin was loaded onto a Superdex 200 column with 200 mM NaCl, 0.1 M Tris (pH 8), 10% glycerol and after the elution was finished, peak fractions were collected and concentrated using VivaSpin 20 (Sartorius).

#### Electrophoretic mobility shift assay (EMSA)

First, the binding reactions containing 25mM Tris-HCl pH 7.5, 100 mM NaCl, 1 mM EDTA, 6% glycerol, 1 mM DTT (dithiothreitol) and 1 nM radiolabelled DNA were mixed with the indicated amounts of FANCD2/FANCI. The reactions were then left at the room temperature for 60-90 min to facilitate the binding of the heterodimer and the radioactive probe. Subsequently, 0.025% BPB (bromophenol blue) was added into each reaction, the samples were loaded into a 4% polyacrylamide (30:1) 0.4xTBE gel and run at 10mA for 3-4h at 4°C. Finally, the gel was fixed and dried for 2h at 80°C and exposed to a photo-stimulable phosphor imager plate.

#### Preparation of radiolabelled DNA substrates

DNA molecules used for EMSA were prepared as described.^16^ The DNA oligos were annealed in a buffer containing 10mM Tris-HCl pH 7.5, 100mM NaCl and 1mM EDTA. ICL14 was created by annealing the following DNA oligonucleotides: ICL14 (+): 5’-CATTGTGAATTCGCCTCTCTGTCTAGCCGAAGCTCGAAACGATCTTGTGC-3’; ICL14 (−): 5’-GTCCATCAAAGTTCGACTGTGCGGCTAGACAGAGAGGCGAATTCACAATG-3’.

#### *In vitro* monoubiquitination assay

The described *in vitro* monoubiquitination assay is a derivative of a previously published method.^40^ Reaction volumes of 25 μl contained 17 nM UBA1, 0.64 mM UBE2T, 4.2 mM 6xHis-Ub, 0.37 mM FANCL, 0.25 mM FANCD2/FANCI or its mutants and 20mM pBlueScript SKII(+) in the following reaction buffer: 50 mM Tris (pH 7.5), 100 mM KCl_2_, 2 mM MgCl_2_, 0.5 mM DTT and 2 mM ATP. Reactions were vortexed and incubated at room temperature for the indicated times, negative control reactions were incubated for the longest indicated time but contained no ATP. To terminate the reactions, 6x SDS loading buffer supplemented with DTT to a final concentration of 50 mM was used. The samples were loaded on a 5% SDS-PAA gel and subjected to Coomassie brilliant blue staining.

#### Live-cell imaging using confocal microscopy

Live cell imaging experiments were carried out using OLYMPUS IX81 microscope connected to PerkinElmer UltraView Vox spinning disk system equipped with a Plan-Apochromat 60x/1.4 oil objective, the software in use for image capturing was Volocity software 6.3. Stable temperature and CO_2_ concentration during the recordings was maintained using a live cell environmental chamber (Tokai hit). The excitations for EGFP and mCherry were 488nm and 561nm, respectively. Confocal image series were recorded with a frame size of 512x512 pixels and a pixel size of 139 nm. For localized introduction of interstrand crosslinks, cells were plated onto glass bottom dishes (MatTek) and incubated with 20 μg/ml 4,5’,8-trimethylpsoralen (TMP) for 30-60 min at 37°C. Subsequent microirradiation was performed using the FRAP preview mode of the Volocity software by scanning (100 ms for each irradiation time) a preselected area within the nucleus with 65 cycles of the 405nm laser which was set to 1.12 mW per cycle. The intensities of both mCherry and EGFP at microirradiated sites were quantified using ImageJ with Fiji and normalized by their intensities before microirradiation.

#### Live-cell single-molecule tracking analysis

Cells were plated on gelatinised 35mm glass-bottom petri dishes (MatTek) at least 12 hours before imaging. Cells were labelled in 5 nM Jenelia Fluor 549 (JF-549) for 30-60 minutes and washed twice with PBS and one time with colorless DMEM (D7777, Sigma Aldrich) supplemented with 10% FBS, 2mM L-Glutamine (G7513, Sigma Aldrich) for 5 minutes in a 5% CO_2_ incubator. 20 mM HEPES pH7.5 were supplemented to the medium before imaging. Single-molecule tracking experiments were conducted on a custom-built PALM/TIRF (Photoactivated Localization Microscopy/Total Internal Reflection Fluorescence) microscope with a highly inclined and laminated optical sheet (HILO) microscopy setting.^36^ The best HILO angle was found before each experiment by maximizing the brightness of the fluorescent spots in the chosen focal plane. The sample temperature during the imaging was maintained at 37°C by using a heated stage. For determining different cell phases, the absence or presence of EGFP-1-110-Geminin in nuclei was empirically evaluated by inspecting cells using 0.5% 488 nm excitation laser with a GFP channel filter. For tracking of diffusing Halo-FANCD2 molecules, movies were recorded with continuous ∼10% 561 nm laser excitation. To introduce ICL damage in cells, 20% 405 nm laser illumination was used to treat cells for 20 seconds and imaging was performed post-irradiation after 15 minutes incubation. Movies of 2,000-4,000 frames were acquired at 20 ms per frame using a 256x256 pixels (0.096 μm/pixel) region of interest. Fixed-cell single-molecule tracking analysis was performed as follows. Cells were plated, labeled with JF-549, and washed as previously described. After washing, cells were fixed with 4% paraformaldehyde in PBS for 1 hour at room temperature and washed twice with PBS. Subsequently, cells were covered with colorless DMEM with supplements. Imaging was performed in the same way as for live cells.

The obtained movies were quantified in MATLAB.^36^ Further data processing and statistical analysis were performed using Excel and GraphPad Prism 8. Positions of fluorescent molecules were detected in each frame using thresholding of band pass-filtered images, followed by localization using a phasor algorithm.^41^ Localizations were linked to tracks if they remain within a circular region of 8 pixels radius in consecutive frames. A tracking memory parameter of 1 frame allowed for blinking or missed detection of a molecule. The mean diffusion coefficient (D) for each track was calculated from the mean squared displacement (MSD): D = MSD/(4·Δt), with Δt = 20 ms. Tracks with less than 4 steps were discarded from the analysis. The population of bound molecules was quantified based on the fraction of tracks with D ≤ 0.3 μm^2^/s.

### Quantification and statistical analysis

Statistical parameters, including statistical tests used, number of events quantified, standard error of the mean, and statistical significance are reported in the figures and in the figure legends. Statistical analysis has been performed using Microsoft Office Excel software and statistical significance is determined by the value of p < 0.05.

## Data Availability

• All data reported in this paper will be shared by the lead contact upon request. • This study did not generate original code. • Any additional information required to reanalyze the data reported in this paper is available from the lead contact upon request.

## Supplemental information

Document S1. Figures S1–S4

Document S2. Article plus supplemental information
