# Supplementary material for: Phosphorylation by ATR triggers FANCD2 chromatin loading and activates the Fanconi anemia pathway
Source: Cell Rep. 2023 Jun 30;42(7):112721. doi: 10.1016/j.celrep.2023.112721 (PMC10933773; doi:10.1016/j.celrep.2023.112721)
Supplement: Document S1. Figures S1–S4 [file mmc1.pdf]

Cell Reports, Volume *42*

Supplemental information

Phosphorylation by ATR triggers

FANCD2 chromatin loading

and activates the Fanconi anemia pathway

Marian Kupculak, Fengxiang Bai, Qiang Luo, Yasunaga Yoshikawa, David Lopez-Martinez, Hannan Xu, Stephan Uphoff, and Martin A. Cohn

Figure S1

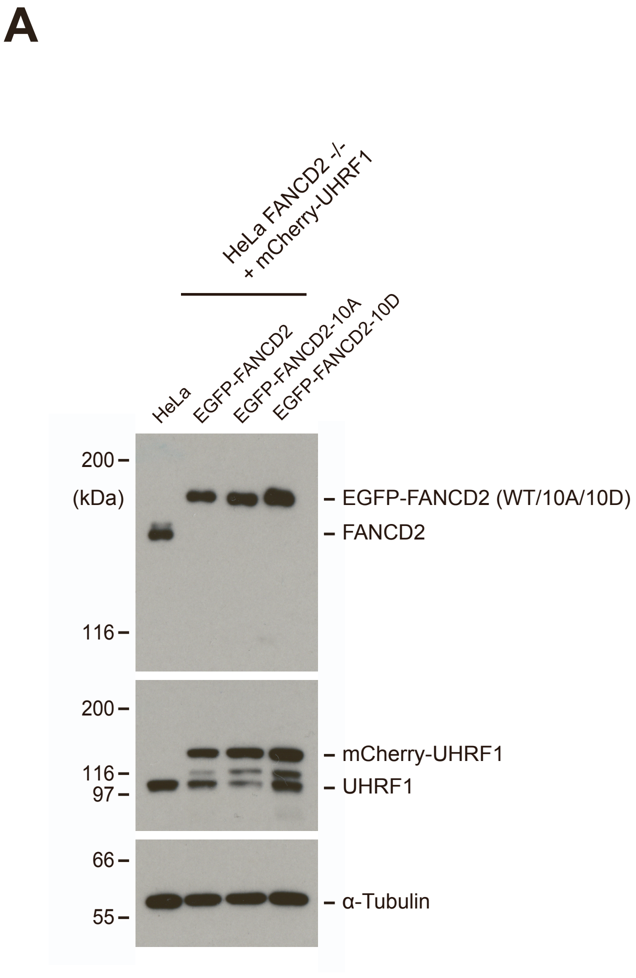

Figure S1 (data relating to Figures 2, 4, 5 and S3). Expression levels of proteins expressed exogenously in HeLa cells. A) Immunoblot analysis of cell lysates from HeLa cells expressing the proteins indicated.

Figure S2

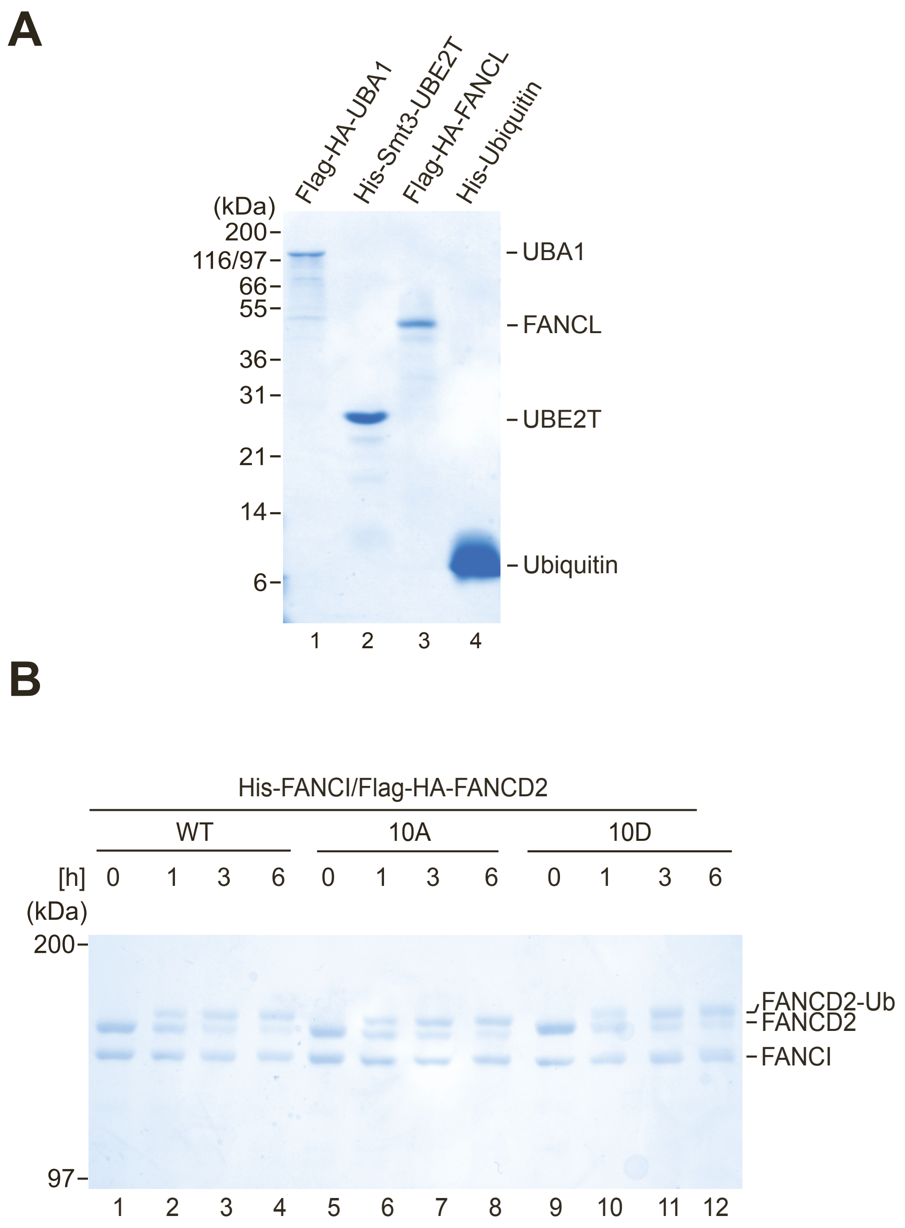

Figure S2 (data relating to Figure 6). *In vitro* ubiquitination of FANCD2 using purified components. A) Proteins purified for the reconstituted ubiquitination reaction. Coomassie brilliant blue stain. B) Ubiquitination assay using FANCD2/FANCI, FANCD2-10A/FANCI FANCD2-10D/FANCI complexes (Figure 6A) as substrates. Coomassie brilliant blue stain.

Figure S3

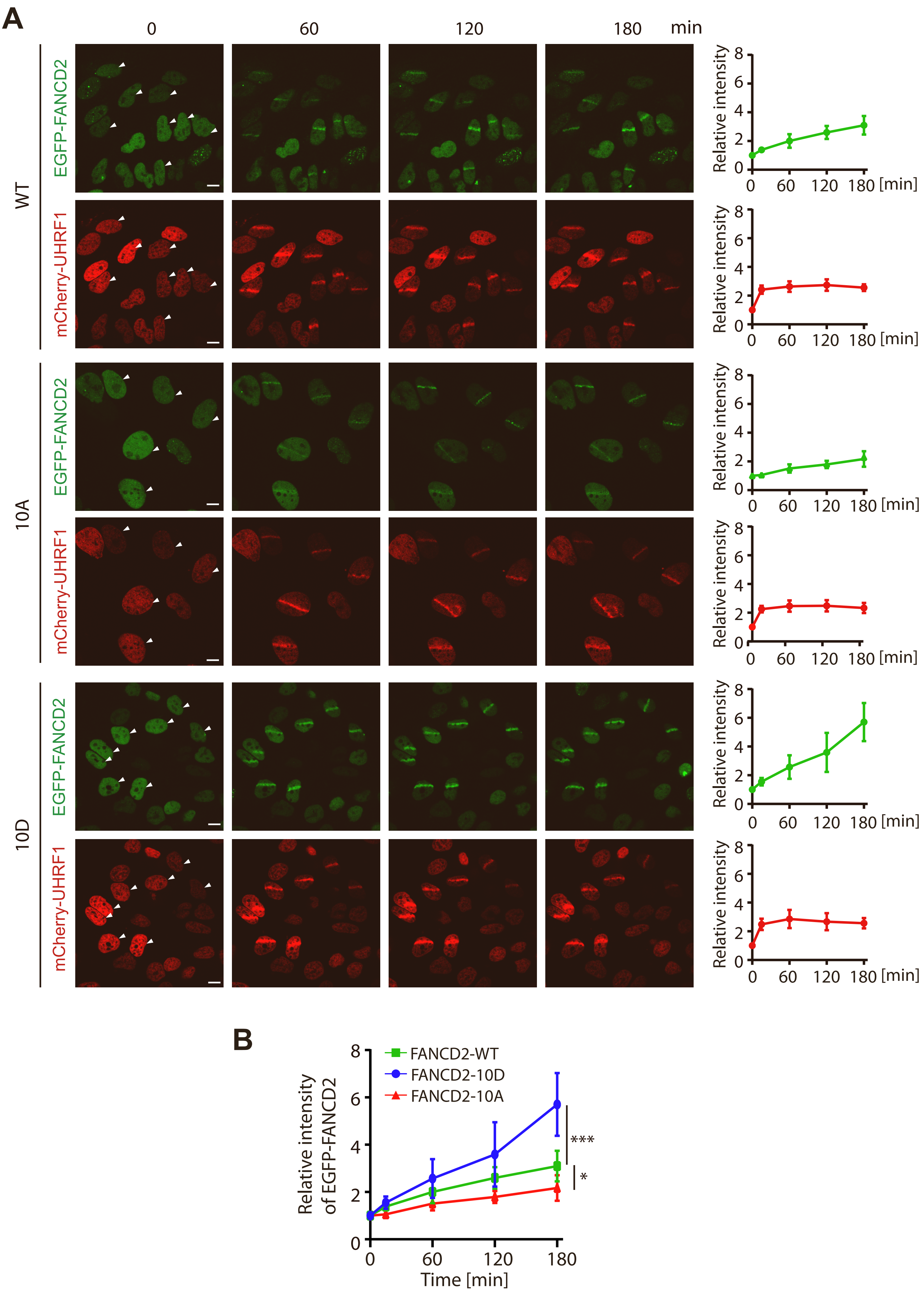
A
0
60
120
180
min
WT
10A
10D
EGFP-FANCD2
mCherry-UHRF1
Relative intensity
0
2
4
6
8
0
60
120
180
[min]
B
FANCD2-WT
FANCD2-10D
FANCD2-10A
Relative intensity of EGFP-FANCD2
Time [min]
***
*

Figure S3 (data relating to Figure 6). Mimicking constitutive phosphorylation of FANCD2 causes augmented recruitment to ICLs. A) Live-cell imaging of HeLa FANCD2-/- complemented with EGFP-FANCD2-WT, EGFP-FANCD2-10A and EGFP-FANCD2-10D. After adding TMP, cells were microirradiated at the specific areas (marked by white arrows) and recorded for the indicated times. mCherry-UHRF1 is used as a control (stripe intensities quantified as mean ± SD; number of cells analyzed: 7 for WT, 5 for 10A, 7 for 10D; scale bar = 10 µm). B) Chart comparing the recruitments of EGFP-FANCD2 for the three analyzed cell lines shown in (A). See also Figure S1.

Figure S4

A

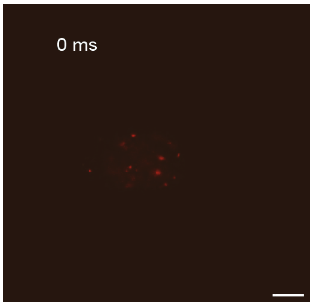

D

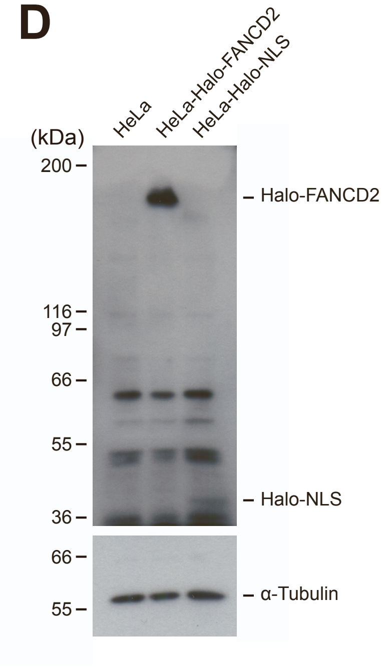

E

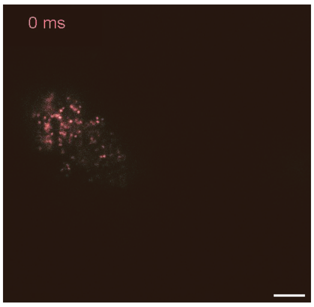

B

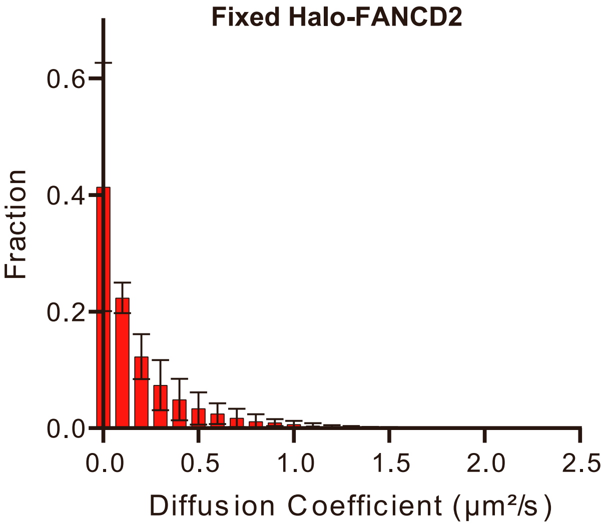

F

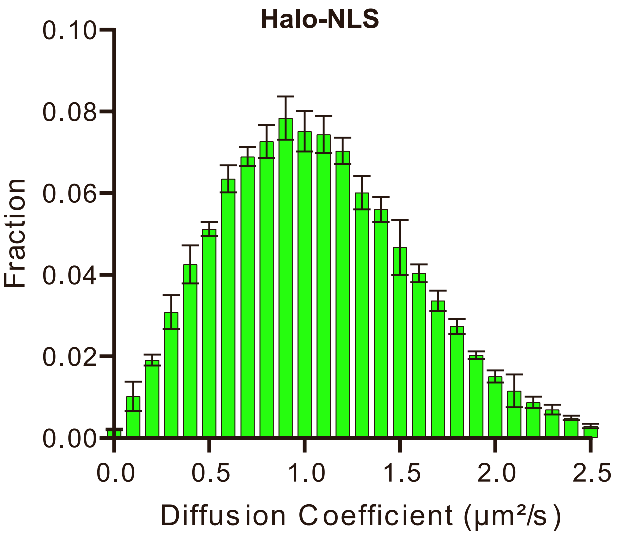

C

	Fixed Halo-FANCD2		Halo-NLS	
Diffusion coefficient ($\mu m^2/s$)	Fraction	SD	Fraction	SD
0	0.41	0.18	0.00	0.00
0.1	0.22	0.02	0.01	0.00
0.2	0.12	0.03	0.02	0.00
0.3	0.07	0.04	0.03	0.00
0.4	0.05	0.03	0.04	0.00
0.5	0.03	0.02	0.05	0.00
0.6	0.02	0.02	0.06	0.00
0.7	0.02	0.01	0.07	0.00
0.8	0.01	0.01	0.07	0.00
0.9	0.01	0.01	0.08	0.01
1	0.01	0.01	0.08	0.00
1.1	0.00	0.00	0.07	0.00
1.2	0.00	0.00	0.07	0.00
1.3	0.00	0.00	0.06	0.00
1.4	0.00	0.00	0.06	0.00
1.5	0.00	0.00	0.05	0.01
1.6	0.00	0.00	0.04	0.00
1.7	0.00	0.00	0.03	0.00
1.8	0.00	0.00	0.03	0.00
1.9	0.00	0.00	0.02	0.00
2	0.00	0.00	0.02	0.00

G

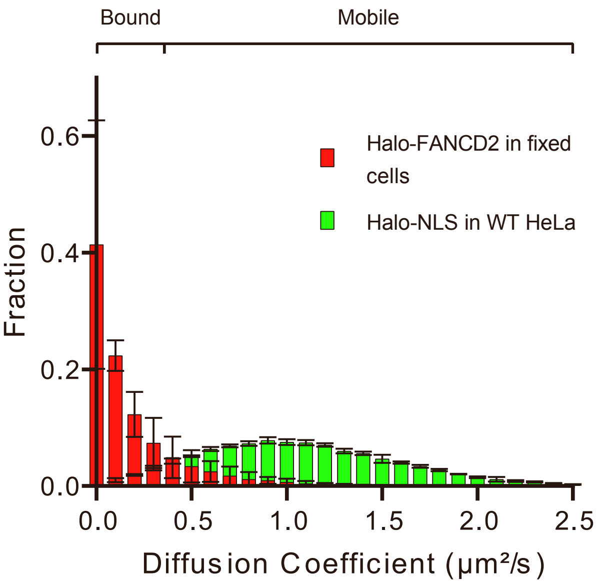

Figure S4 (data relating to Figure 7). Single particle tracking demonstrate how genome-wide loading of FANCD2 to ICLs is licensed by ATR. A) Super-resolution microscopy image shows individual Halo-FANCD2 molecules in fixed HeLa cells. Scale bar = 5 μm. B) Histogram graph of diffusion coefficients for fixed Halo-FANCD2 molecules. Data are means ± SD. C) Diffusion coefficients of FANCD2 molecules in fixed HeLa cells (shown in panels A-B). Cells are fixed with 4% paraformaldehyde in PBS to mimic the bound FANCD2 molecules. (n=8 cells, 4000 frames per cell). Table also shows diffusion coefficients for Halo-NLS (shown in panels E-F). Red figures indicate categories of diffusion coefficients ≤ 0.3 $\mu m^2/s$, relating to molecules classified as being in the bound state. D) Immunoblot analysis of cell lysates of HeLa expressing Halo-FANCD2 (knock-in) or Halo-NLS. E) Super-resolution microscopy image shows individual Halo-NLS molecules HeLa cells. Scale bar = 5 μm. F) Histogram graph of diffusion coefficients for Halo-NLS molecules. Data are means ± SD. G) Distribution of diffusion coefficients of fixed FANCD2 molecules, and free Halo-NLS molecules, which possess at least 5 localizations per track (data shown in panels B, C and F). The data are split into two populations based on the diffusion coefficient per track, bound molecules ($D \leq 0.3\ \mu m^2/s$) and mobile molecules ($D > 0.3\ \mu m^2/s$). (n=4 cells per treatment, > 5,000 tracks per cell). Data are means ± SD.
